# Clinical characteristics and prognostic value of renal immune complex deposition in patients with light chain amyloidosis

**DOI:** 10.3389/fonc.2022.949702

**Published:** 2022-10-13

**Authors:** Jipeng Yan, Di Wang, Jin Zhao, Meilan Zhou, Boyong Huang, Yan Xing, Wei-Feng Guo, Shiren Sun

**Affiliations:** ^1^ Department of Nephrology, Xijing Hospital, Fourth Military Medical University, Xi’an, China; ^2^ Department of Geriatrics, Xijing Hospital, Fourth Military Medical University, Xi’an, China; ^3^ School of Electrical Engineering, Zhengzhou University, Zhengzhou, China; ^4^ State Key Laboratory of Oncology in South China, Collaborative Innovation Center for Cancer Medicine, Sun Yat-sen University Cancer Center, Guangzhou, China

**Keywords:** light chain amyloidosis, immune complexes, prognostic factors, overall survival, renal survival

## Abstract

Although patients with light chain amyloidosis (AL) may present with co-deposition of amyloid and immune complexes (ICs) in renal biopsies, data on clinical characteristics and prognostic value of renal IC deposition are limited. A total of 73 patients with AL amyloidosis who were newly diagnosed by renal biopsy in Xijing Hospital (Xi’an, China) were divided into two groups (IC and non-IC groups). As a result, renal IC deposition was found in 26% of patients. Patients with IC deposition were associated with more urinary protein excretion and lower serum albumin. Notably, patients in the non-IC group achieved higher hematological overall response rate (81.5% *vs.* 47.4%, *p* = 0.007) and ≥VGPR rate (75.9% *vs.* 39.8%, *p* = 0.004) compared with those in IC group. Renal response rate was also higher in the non-IC group (63% *vs.* 31.6%, *p* = 0.031). With the median follow-up time of 19 months, a significantly worse overall survival was observed in patients with the IC group as compared with those without renal IC deposition in the Kaplan–Meier analysis (*p* = 0.036). Further multivariate analysis demonstrated that renal immune complex deposition was associated with worse overall survival in patients with AL amyloidosis (HR 5.927, 95% CI 2.148–16.356, p = 0.001).

## Introduction

Immunoglobulin light chain (AL) amyloidosis is a heterogeneous and life-threatening disease characterized by the deposition of amyloid fibrils derived from misfolded light chains (1). The median survival time of AL amyloidosis is only approximately 6 months in untreated patients, and the high mortality rate has attracted the attention of clinicians (2). The current staging and prognostic classifications for AL amyloidosis use serum levels of cardiac troponins, N-terminal brain natriuretic peptide (NT-proBNP), and the difference between involved and uninvolved free light chains (dFLC) to predict outcomes of patients (3). However, significant heterogeneity in prognosis still exists, and efforts are needed to identify additional underlying prognostic factors that can be used to stratify patients with different prognoses.

In recent years, several biomarkers and factors with prognostic value have been discussed widely. On the one hand, the pattern and extent of organ involvement at baseline are important for the prognosis of patients with AL amyloidosis. The kidney is the most frequently affected organ, and renal AL amyloidosis usually manifests as nephrotic-range proteinuria and progressive worsening of renal dysfunction (4, 5). The combination of proteinuria and estimated glomerular filtration rate (eGFR) has also been used by two staging systems to predict the risk of end-stage renal disease (ESRD) (6). However, the severity of cardiac involvement remains the main prognostic determinant in AL amyloidosis (7). Therefore, most of the organ biomarkers with prognostic relevance to overall survival (OS) in AL represent the cardiac amyloid load (septum thickness), destruction of cardiomyocytes (troponins), or cardiac dysfunction (left ventricular ejection fraction and NT-proBNP/BNP) (8–12). More involved organs result in a worse OS (13). On the other hand, in the area of plasma cellular factors, serum-free light chain (FLC) and dFLC have proven effectiveness, and their prognostic significance for OS in AL amyloidosis has been validated by several studies (3, 13–15). A higher plasma cellular burden in bone marrow cytology or histology and interphase fluorescence *in situ* hybridization (iFISH) abnormalities are also independent prognostic factors in AL amyloidosis (16–19). Finally, hematological and organ responses to plasma cell-directed therapy are strongly and directly associated with survival in AL patients (20, 21). Given all the prognostic factors mentioned above, we found that although the kidney is the most frequently affected organ, the major determinant of outcome in amyloidosis is the extent of cardiac involvement, and overlooking the pathological features of renal biopsy in AL amyloidosis is very common.

Based on immunofluorescence in the pathology of clinical renal biopsy, we found that a small proportion of patients with AL amyloidosis may present with co-deposition of amyloid and immune complexes in renal biopsies. However, the clinical and prognostic significance of the renal immune complexes in patients with AL amyloidosis remains unclear. There are few studies about IC deposits in renal biopsies of patients with AL amyloidosis. Therefore, this study was conducted to explore the prevalence and significance of renal immune complex deposition in patients with AL amyloidosis.

## Materials and methods

### Patients

Patients with systemic AL amyloidosis who were newly diagnosed by renal biopsy in Xijing Hospital (Xi’an, China) from December 2015 to March 2021 were included in our study. The diagnosis of systemic AL amyloidosis was confirmed by the presence of apple-green birefringence under polarized light after Congo red staining and the identification of fibrils with a diameter of 8–12 nm by electron microscopy (22). Patients who suffered from diabetes, membranous nephropathy, IgA nephropathy, or other confounding diseases based on relevant clinical and pathological information were excluded. Clinical and pathological data of patients meeting the inclusion criteria were retrospectively collected and analyzed, including a brief history, demographic information, clinical manifestations, important laboratory tests and imaging examinations, renal histological data, treatment, and long-term follow-up. The study was approved by the Xijing Hospital Ethics Committee.

### Renal histology

Renal biopsy specimens were examined by routine light microscopy (LM), immunofluorescence (IF) and electron microscopy (EM). For IF, we applied the direct immunofluorescent method to detect IgG, IgA, IgM, C3, C4, C1q, λ and κ. In detail, 4 μm frozen sections were placed at room temperature for 30 minutes. Then we added the fluorescein isothiocyanate (FITC)-labeled anti-human antibodies (DAKO, **Supplementary Table**) and stay overnight at 4 °C. After that, we washed the sections with PBS for 2-3 minutes, and then dry them at room temperature. Finally, we used glycerin for sealing. The results were graded on a scale of 0 to 3+ according to the intensity of the fluorescence. Deposition of immune complexes was defined as a score of 1+ or higher staining for any kind of Ig. In this study, there were no other known secondary diseases by the confirmation of our pathologist according to clinical history, laboratory examinations, and pathological results. Furthermore, the laser microdissection combined with mass spectrometry (LMD-MS), the gold standard technique to type the amyloid (23, 24), was done to confirm the amyloid type and exclude heavy or heavy/light chain amyloidosis.

### Laser microdissection and mass spectrometry-based analysis

The methods have previously been published (24–26). In brief, for each case, a 10-μm paraffin section was stained with Congo Red, and then amyloid deposits were identified under fluorescent light and microdissected with LMD. Each microdissection contained an area of 50,000 to 60,000 μm^2^, and two to four separate microdissections were analyzed for each specimen. Microdissected fragments were digested into tryptic peptides and analyzed by liquid chromatography–electrospray tandem MS. MS raw data files were queried by the use of three different algorithms (Mascot, Sequest, and X!Tandem), the results were assigned peptide and protein probability scores, and they were displayed by the use of Scaffold (Proteome Software). For each case, a list of proteins based on peptides identified by MS was generated.

### Follow-up and outcome evaluation

The Mayo 2012 Clinic Staging System (3) and the National Amyloidosis Centre guidelines (27) were used to evaluate the disease risk stratification and organ involvement, respectively. Hematological and organ responses were defined according to the 2012 updated AL amyloidosis response criteria (28) and were documented as the best responses achieved prior to disease progression or death. Renal impairment staging was defined using the criteria from the published article (6).

### Statistical analysis

Statistical analysis was performed using the SPSS software package (version 20.0, IBM, Armonk, New York). Data are expressed as number (%) for qualitative variables and as mean ± SD or median (interquartile range, IQR) for quantitative variables. Student’s t-test, the Kruskal–Wallis-test or the χ2 test was used to compare the differences in variables between non-IC and IC groups. Overall survival was defined as the time from initial diagnosis by renal biopsy at our center to death or last follow-up. Overall survival and renal survival were analyzed using the Kaplan-Meier method, and curves were compared with the log-rank test. Cox proportional hazards regression analysis was used to identify independent prognostic factors for OS, which are reported with their hazard ratios (HRs) and 95% CIs. Variables with p < 0.05 in univariate analyses were entered into the multivariate model. Unadjusted and adjusted p values were generated by univariate and multivariate cox regression analysis, respectively. The between-group difference was tested at the two-sided alpha level of 0.05. All p values were two-sided.

## Results

### Kidney biopsy findings in light chain amyloidosis with immune complex deposition

**Figure 1:**
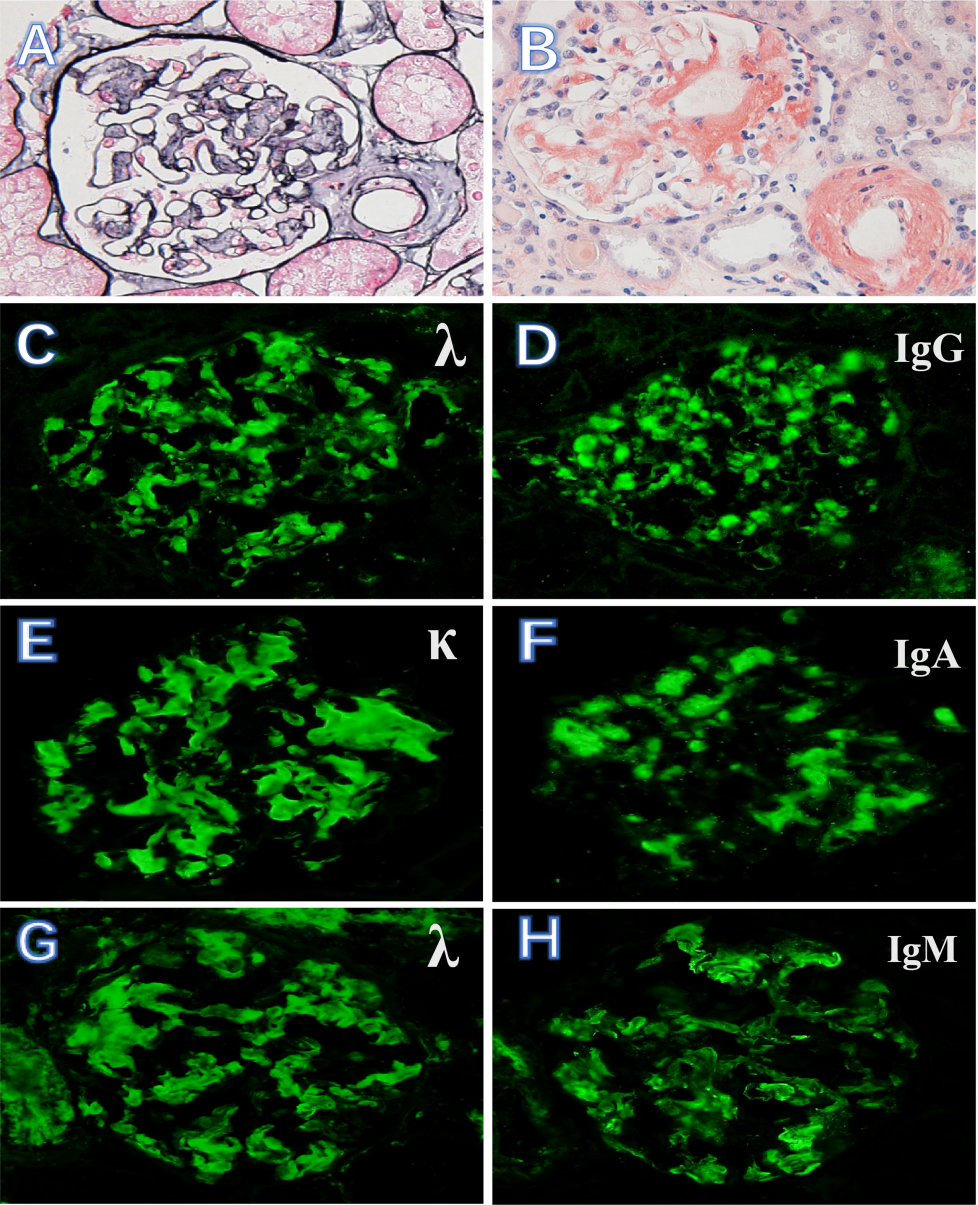
Kidney biopsy findings in AL amyloidosis with immune complex deposition. **(A, B)** Massive amyloid deposits are present in glomeruli and arterioles (PASM, ×400) and as brick-red in Congo red stain (×400). **(C, D)** AL amyloidosis with λ light chain-restricted staining and Ig G++. **(E, F)** κ light chain-restricted staining and Ig A++. **(G, H)** λ light chain-restricted staining and Ig M++ (immunofluorescence, ×400).

The biopsy specimen demonstrates positive staining with Congo red dye and characteristic apple-green birefringence observed with polarized light. The amyloid deposits were localized in glomeruli, arterioles, and tubulointerstitium. There is a nodular appearance due to amorphous, acellular eosinophilic pale material (**Figures 1A, B**). Of the study cohort, 19 patients (26%) had renal immune complex deposition. Specific results of immunofluorescence and mass spectrometric analysis of these patients are listed in **Table 1**. The types of Ig presented in the deposits were as follows: IgG in 9 cases (**Figures 1C, D**), IgA in 6 cases (**Figures 1E, F**), IgM in 14 cases (IgG + IgA +IgM in 4 cases, IgG + IgM in 1 case, IgA + IgM in 2 cases, **Figures 1G, H**), respectively. Electron microscopy shows non-branching 8~12-nm diameter fibrils with random orientation. Amyloid fibrils coexisted with electron-dense deposits in the expansion of the mesangium.

**Table 1:** Immunofluorescence and mass spectrometric analyses of renal biopsy specimens from 19 patients with immune complex deposition.

Number	Immunofluorescence								Mass spectrometry
	λ	κ	IgG	IgA	IgM	C3	C4	C1q	
1	++	−	++	+	+	+	−	±	AL-λ
2	+++	−	+++	−	−	−	−	++	AL-λ
3	−	++	+	+	+	−	−	++	AL-κ
4	++	−	−	−	++	++	−	−	AL-λ
5	+++	−	++	−	−	−	−	++	AL-λ
6	+++	−	−	−	++	−	−	−	AL-λ
7	++	−	−	−	++	+	−	++	AL-λ
8	+++	−	−	−	++	−	−	−	AL-λ
9	+++	−	++	−	−	++	−	−	AL-λ
10	++	−	−	−	+++	−	−	−	AL-λ
11	++	−	−	−	++	−	−	+++	AL-λ
12	+++	−	−	−	+++	−	++	++	AL-λ
13	++	−	−	−	++	−	+	+	AL-λ
14	+++	−	++	−	−	++	−	++	AL-λ
15	++	−	−	++	++	+	−	−	AL-λ
16	++	−	++	++	+	++	+	−	AL-λ
17	++	−	++	−	++	−	++	++	AL-λ
18	++	−	−	++	++	−	−	−	AL-λ
19	+++	−	++	++	++	+	−	+	AL-λ

The results were graded on a scale of 0 to 3+ (-, +, ++, +++) according to the intensity of the fluorescence.

### Baseline and disease characteristics of patients in the study cohort

A total of 73 patients with biopsy-proven renal AL amyloidosis in our clinic were included in the final analysis. Patient demographic and disease characteristics in the whole group and divided by renal IC deposit presence are presented in **Table 2**. The median age of all patients at the first visit was 59 years (IQR, 51–65), with 45 (61.6%) men. More than half of the patients (78.1%, n = 57) had ≥2 organs involved, with 78.1% and 8.2% having cardiac and hepatic involvement, respectively. The majority of patients (91.8%, n = 67) had lambda (λ) isotype. The median dFLC was 100 mg/L (IQR, 41–148) for all patients. Patients with renal IC deposition had higher proteinuria (4.5 *vs.* 2.9 g/day, *p* = 0.047) and lower blood albumin (22.4 *vs.* 24.7 g/L, *p* = 0.033) than patients without IC deposits. For renal impairment stage in patients who had available data of urine total protein and eGFR, there were 48 patients (68.5%) with stage I, 20 patients (27.4%) with stage II, and only 5 patients (6.8%) with stage III. In terms of the Mayo 2012 staging system, 22 patients (30.1%) had Mayo stage I, 21 (28.8%) had Mayo stage II, 22 (30.1%) had Mayo stage III, and 8 (11%) had Mayo stage IV. When it comes to the first-line treatment between the two groups, no significant difference was seen between the two groups. Specifically, the percentages of BD (bortezomib and dexamethasone), CTD (cyclophosphamide, thalidomide, and dexamethasone), and MD (melphalan and dexamethasone) regimens in the IC group were 36.8%, 31.6%, and 26.3%, respectively, while those in the non-IC group were 46.3%, 18.5%, and 25.9%, respectively.

**Table 2:** Baseline characteristics between the IC group and the non-IC group.

Variable	All patients (N = 73)	Non-IC group (n = 54)	IC group (n = 19)	*p-*Value (non-IC *vs.* IC)
Age, years, median (IQR)	59 (51–65)	59 (50–66)	57 (52–64)	0.712
Male sex, N (%)	45 (61.6%)	37 (68.5%)	8 (42.1%)	0.056
Organ involved, N (%)				
≥2 organ	57 (78.1%)	41 (75.9%)	16 (84.2%)	0.537
Renal	73 (100%)	54 (100%)	19 (100%)	1.000
Cardiac	57 (78.1%)	41 (75.9%)	16 (84.2%)	0.537
Hepatic	6 (8.2%)	4 (7.4%)	2 (10.5%)	0.647
Lambda restricted, N (%)	67 (91.8%)	49 (90.7%)	18 (94.7%)	1.000
dFLC, mg/ml, median (IQR)	100 (41–148)	103 (41–146)	83 (41–162)	0.745
Proteinuria, g/day, median (IQR)	3.1 (1.5–4.7)	2.9 (1.3–4.5)	4.5 (2.2–7.5)	**0.047**
eGFR, ml/min/1.73 m^2^, median (IQR)	75 (62–102)	76 (62–99)	75 (60–126)	0.674
Alb, g/L, median (IQR)	24.2 (19.1–32.5)	24.7 (19.2–33.7)	22.4 (18.3–24.7)	**0.033**
NT-proBNP, pg/ml, median (IQR)	1,059 (360–4,300)	1,805 (424–4,518)	671 (335–2,459)	0.795
Troponin T, ng/ml, median (IQR)	0.029 (0.011–0.074)	0.032 (0.01–0.083)	0.026 (0.015–0.064)	0.776
Alkaline phosphatase, U/L (IQR)	77 (58–97)	77 (57–96)	73 (60–101)	0.614
LVEF (%)	57 (55–59)	57 (55–60)	58 (55–59)	0.642
IVST (mm)	11 (10–12)	11 (9–13)	11 (10–12)	0.804
Renal impairment stage, no. (%)				0.197
Stage I, n (%)	48 (68.5%)	37 (68.5%)	11 (57.9%)	
Stage II, n (%)	20 (27.4%)	15 (27.8%)	5 (26.3%)	
Stage III, n (%)	5 (6.8%)	2 (3.7%)	3 (15.8%)	
Mayo 2012 stage, N (%)				0.436
Stage I, n (%)	22 (30.1%)	15 (27.8%)	7 (36.8%)	
Stage II, n (%)	21 (28.8%)	14 (25.9%)	7 (36.8%)	
Stage III, n (%)	22 (30.1%)	19 (35.2%)	3 (15.8%)	
Stage IV, n (%)	8 (11%)	6 (11.1%)	2 (10.5%)	
First-line treatment, n (%)				0.647
BD	32 (43.8%)	25 (46.3%)	7 (36.8%)	
MD	16 (21.9%)	10 (18.5%)	6 (31.6%)	
CTD	19 (26%)	14 (25.9%)	5 (26.3%)	
Other	6 (8.2%)	5 (9.3%)	1 (5.3%)	

Bold values indicate statistical significance at *p* < 0.05. IC, immune complex; IQR, interquartile range; dFLC, difference in the involved to uninvolved free light chain; eGFR, estimated glomerular filtration rate; Alb, albumin; NT-proBNP, N-terminal brain natriuretic peptide; LVEF, left ventricular ejection fraction; IVST, inter-ventricular septum thickness; BD, bortezomib + dexamethasone; CTD, cyclophosphamide + thalidomide + dexamethasone; MD, melphalan and dexamethasone.

### Best hematological and organ responses in patients with and without renal immune complex deposition

The hematological and organ response rates in the IC and non-IC groups are described in **Table 3**. The median follow-up times were 16.5 and 20.5 months for the IC group and non-IC group, respectively. Patients in the non-IC group had a higher overall hematological response (OHR) rate than those in the IC group (81.5% *vs.* 74.4%, *p* = 0.007). There were no differences in complete response (CR) rate (46.3% *vs.* 26.3%, *p* = 0.269) or very good partial response (VGPR) rate (29.6% *vs.* 10.5%, *p* = 0.207) between the non-IC group and IC group. However, a significant difference was seen in the ≥VGPR rate between the non-IC group and IC group (75.9% *vs.* 39.8%, *p* = 0.004).

**Table 3:** Best hematological and organ response between the IC group and the non-IC group.

	All patients (n = 73)	Non-IC group (n = 54)	IC group (n = 19)	*p-*Value (non-IC *vs.* IC)
Hematologic response, n (%)				
Any response	53 (72.6%)	44 (81.5%)	9 (47.4%)	**0.007**
CR	30 (41.1%)	25 (46.3%)	5 (26.3%)	0.269
VGPR	18 (24.7%)	16 (29.6%)	2 (10.5%)	0.207
PR	5 (6.8%)	3 (5.6%)	2 (10.5%)	0.593
NR	20 (27.4%)	10 (18.5%)	10 (52.6%)	**0.014**
≥VGPR	48 (65.8%)	41 (75.9%)	7 (39.8%)	**0.004**
Renal response, n (%)	40 (54.8%)	34 (63%)	6 (31.6%)	**0.031**
Cardiac response, n (%)	22 (38.6%) (n = 57)	19 (46.3%) (n = 41)	3 (18.8%) (n = 16)	0.073
Hepatic response, n (%)	1 (20%) (n = 5)	1 (33.1%) (n = 3)	0 (0%) (n = 2)	1.000

CR, complete response; VGPR, very good partial response; PR, partial response; NR, no response.

Bold values indicate statistical significance at p<0.05.

Organ response assessment required the involvement of the organ at the moment of treatment onset. A total of 73, 57, and 5 patients were eligible for renal, cardiac, and hepatic response assessments, respectively (**Table 3**). The renal response rate was higher in the non-IC group compared with the IC groups (63% *vs.* 31.6%, *p* = 0.0.031). However, the cardiac and hepatic responses in the non-IC group were numerically higher than those in the IC group, but without significant differences (46.3% *vs.* 18.8%, *p* = 0.073; 33.1% *vs.* 0%, *p* = 1.000).

### Overall survival and renal survival between the non-immune complex group and immune complex group

**Figure 2:**
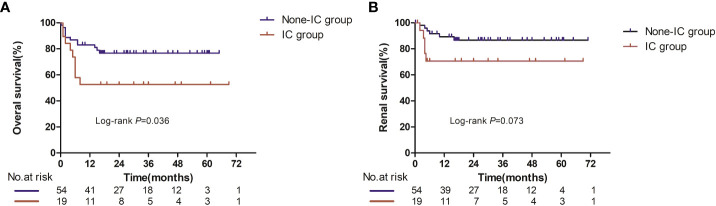
Overall survival **(A)** and renal survival **(B)** in evaluable patients between the IC and non-IC group. Number at risk at certain time points is shown in panel below graph.

During the median follow-up time of 20.5 months for the non-IC group and 16.5 months for the IC group, 21 patients died (12 in the non-IC group and 9 in the IC group). All of the deaths were deemed disease progression. Median OS was not reached in either group based on the Kaplan–Meier survival analysis, but there was a significant difference between the two groups (*p* = 0.036, **Figure 2A**). Patients in the IC group had worse prognoses in overall survival compared with the non-IC group.

As for renal survival, a total of 11 patients had progression to ESRD or the need for dialysis (six in the non-IC group and nine in the IC group). The median renal survival was also not reached in either group (**Figure 2B**), and no significant difference was seen in renal survival between the two groups (*p* = 0.073).

Univariate and multivariate Cox analyses were further performed to identify prognostic factors for OS (**Table 4**). In the univariate Cox regression model, the following variables were significantly associated with OS: renal IC deposition, number of involved organs, alkaline phosphatase (ALP), eGFR, and ≥Mayo 2012 stage III. However, when multivariate analysis was performed, four factors remained: renal IC deposition (HR 5.927, 95% CI 5.148–16.356, *p* = 0.001), ALP (HR 1.005, 95% CI 1.000–1.009, *p* = 0.042), eGFR (HR 0.965, 95% CI 0.936–0.995, *p* = 0.021), and Mayo 2012 stage III/IV (HR 9.903, 95% CI 3.074–31.905, *p* = 0.021) were prognostic predictors of OS in AL amyloidosis.

**Table 4:** Univariate and multivariate Cox regression analyses for overall survival in the study cohort.

	Univariable	Multivariate
Variable	HR (95% CI) *p*	HR (95% CI) *p*
Renal IC deposition	2.424 (1.019–5.763) **0.045**	5.927 (2.148–16.356) **0.001**
Age	0.991 (0.947–1.038) 0.708	Not included
Male sex	1.092 (0.452–2.636) 0.845	Not included
No. of involved organs	2.471 (1.401–4.358) **0.002**	1.668 (0.684–4.067) 0.260
ALP	1.003 (1.001–1.006) **0.019**	1.005 (1.000–1.009) **0.042**
eGFR ≥Mayo 2012 stage III	0.979 (0.964–0.995) **0.009** 6.054 (2.210–16.587) **0.001**	0.965 (0.936–0.995) **0.021** 9.903 (3.074–31.905) **< 0.001**

IC, immune complex; ALP, alkaline phosphatase; dFLC, difference in the involved to uninvolved free light chain; eGFR, estimated glomerular filtration rate; HR, hazard ratio.

Bold values indicate statistical significance at p < 0.05.

## Discussion

Immunoglobulin light chain amyloidosis is an uncommon disorder, characterized by the deposition of Congo-red-positive fibrillary material leading to various impairments in organ function (29). Overall, the prognosis of AL patients is determined by several parts, such as the biology of underlying clonal disease, the pattern and extent of organ involvement at baseline, and the response to treatment (30). With the development of novel agents and their combined application in treatment, the outcomes of patients with AL amyloidosis have greatly improved (31, 32). However, outcomes are still poor when there is cardiac involvement because the major determinant of outcome in amyloidosis is the extent of cardiac involvement (33). Therefore, overlooking the pathological features of renal biopsy in AL amyloidosis is common and may also explain the scant literature on the prevalence and prognostic value of renal IC deposition in AL amyloidosis.

To our best knowledge, this is the first study to comprehensively evaluate the prevalence and prognostic significance of renal immune complex deposition in patients with AL amyloidosis. Data from our study demonstrate that less than one-third of newly diagnosed patients had renal IC deposition, which had worse hematological and renal responses. Renal IC deposition was also associated with worse overall survival, which was further confirmed in the multivariable analysis. However, the mechanism that how IC deposition develops in AL amyloidosis is unclear. O’Nuallain et al. (34) have reported that human sera contain high-affinity antibodies specific for fibrils formed from different amyloidogenic precursor proteins, and they suggested that fibril-reactive IgG Abs may be a novel diagnostic and therapeutic modality to improve the invariably poor prognoses of patients with the amyloid-associated disease. Therefore, we boldly hypothesized that humoral immunity is involved in the pathogenesis of AL amyloidosis, but further studies are needed to confirm it. Exposure to concurrent or secondary infections following immune system perturbation or immunosuppressive therapy and the subsequent abuse of antibiotics can also explain the occurrence of circulating ICs and their deposition in renal tissues (35).

In our study, renal IC deposition was found to be an independent prognostic predictor of overall survival. Several potential hypotheses may explain this phenomenon. First, patients with ICs in glomeruli had higher levels of proteinuria than those without ICs. Higher urinary protein excretion is reported to be an independent risk factor for OS and has also been confirmed to be a clinical indicator to predict progression to dialysis in AL amyloidosis (9). Therefore, it is reasonable to speculate that the deposition of ICs will more or less affect the overall renal survival of patients with renal AL amyloidosis. Although the Kaplan–Meier renal survival analysis showed no significant difference between the two groups (*p* = 0.073), the short median follow-up times (16.5 *vs.* 20.5 months) between the IC and non-IC groups may explain these results. Second, a significant difference in the best hematological and renal response between the two groups was another important finding in our research. Anti-plasma cellular therapy is the cornerstone of treatment in AL amyloidosis since the light chain is the precursor of the amyloid protein. Emerging evidence had proved that hematological response is strictly and directly associated with survival. A 50% decrease in involved free light chain (iFLC) as well as dFLC upon therapy was found to be associated with significantly improved OS (36, 37). To achieve organ response in a fraction of patients, a decrease of dFLC below 40 mg/L should be the minimal goal of therapy (28). Thus, poor hematological and organ responses may account for the higher mortality rate in patients with renal IC deposition. In our study, more patients achieved VGPR or better hematological response and renal response in the non-IC group might result in a better prognosis in overall survival.

The main limitations of this study are related to the biases inherent to its retrospective design. Due to renal biopsy contraindications such as bleeding and kidney atrophy, some patients who only underwent abdominal fat biopsy at diagnosis were not included in our study. Therefore, it was impossible to judge whether these patients had renal immune complex deposition. If the biopsy is small, with only one or a few glomeruli available for examination, immune complex deposition may remain undetected in such biopsies (38). In addition, because of the small sample size and other confounding factors, the present data need validation in both other large retrospective cohorts and prospective trials. Moreover, although the treatments in the two groups did not reach statistical significance, the impact of different treatments on prognosis cannot be ignored. Furthermore, the long-term prognosis could not be evaluated because of the short duration of the follow-up and the small size of the study sample. More meaningful results might be observed with longer follow-up times.

In conclusion, this study demonstrated that less than one-third of patients with AL amyloidosis had renal IC deposition, which is associated with worse hematological response and renal response. Notably, the deposition of immune complexes in the kidney indicates a significantly worse prognosis of overall survival in AL amyloidosis. Additional research is required to ascertain the etiology of IC deposition in the kidney. Our research has solved the problems of renal IC deposition encountered in practical clinical work and may further promote the utility of renal IC deposition in combination with other known predictors of mortality to improve the staging system in patients with AL amyloidosis.

## Data availability statement

The raw data supporting the conclusions of this article will be made available by the authors, without undue reservation.

## Ethics statement

Written informed consent was obtained from the individual(s) for the publication of any potentially identifiable images or data included in this article.

## Author contributions

JY, SS, and DW conceived and designed this study. JZ, MZ, BH, W-FG, and YX participated in data acquisition and analysis. JY drafted the manuscript. All authors contributed to the article and approved the submitted version.

## Funding

This study was sponsored by grants from the Xijing Hospital discipline promoting plan (reference number: XJZT18MDT17) and National Natural Science Foundation of China grants (reference number: 81870470).

## Acknowledgments

We thank American Journal Experts (AJE) for their linguistic assistance during the preparation of this manuscript.

## Conflict of interest

The authors declare that the research was conducted in the absence of any commercial or financial relationships that could be construed as a potential conflict of interest.

## Publisher’s note

All claims expressed in this article are solely those of the authors and do not necessarily represent those of their affiliated organizations, or those of the publisher, the editors and the reviewers. Any product that may be evaluated in this article, or claim that may be made by its manufacturer, is not guaranteed or endorsed by the publisher.
