# Supplementary material for: Clinical characteristics and prognostic value of renal immune complex deposition in patients with light chain amyloidosis
Source: Front Oncol. 2022 Oct 13;12:949702. doi: 10.3389/fonc.2022.949702 (PMC9608106; doi:10.3389/fonc.2022.949702)
Supplement: Supplementary file 1 [file Table_1.docx]

Supplementary Material

# Supplementary Tables

**Supplementary table 1.** Antibodies used in the direct immunofluorescent method.

| **Number** | **Product name** | **Specifications** | **Code Number** | **Supplier** |
| --- | --- | --- | --- | --- |
| 1 | FITC-labeled anti human IgG | 2ml | F0202 | DAKO |
| 2 | FITC-labeled anti human IgA | 2ml | F0204 | DAKO |
| 3 | FITC-labeled anti human IgM | 2ml | F0203 | DAKO |
| 4 | FITC-labeled anti human C3c | 2ml | F0201 | DAKO |
| 5 | FITC-labeled anti human C4c | 2ml | F0169 | DAKO |
| 6 | FITC-labeled anti human C1q | 2ml | F0254 | DAKO |
| 8 | FITC-labeled anti human Kappa | 2ml | F0198 | DAKO |
| 9 | FITC-labeled anti human Lambda | 2ml | F0199 | DAKO |
